# HLA-DQA1 expression is associated with prognosis and predictable with radiomics in breast cancer

**DOI:** 10.1186/s13014-023-02314-4

**Published:** 2023-07-11

**Authors:** JingYu Zhou, TingTing Xie, HuiMing Shan, GuanXun Cheng

**Affiliations:** Department of Radiology, Peking University Shenzhen Hospital, LianHua Road, Shenzhen, 518000 Guangdong China

**Keywords:** Breast cancer, Biomarker, Prognosis, Human leukocyte antigen, Radiomics

## Abstract

**Background:**

High HLA-DQA1 expression is associated with a better prognosis in many cancers. However, the association between HLA-DQA1 expression and prognosis of breast cancer and the noninvasive assessment of HLA-DQA1 expression are still unclear. This study aimed to reveal the association and investigate the potential of radiomics to predict HLA-DQA1 expression in breast cancer.

**Methods:**

In this retrospective study, transcriptome sequencing data, medical imaging data, clinical and follow-up data were downloaded from the TCIA (https://www.cancerimagingarchive.net/) and TCGA (https://portal.gdc.cancer.gov/) databases. The clinical characteristic differences between the high HLA-DQA1 expression group (HHD group) and the low HLA-DQA1 expression group were explored. Gene set enrichment analysis, Kaplan‒Meier survival analysis and Cox regression were performed. Then, 107 dynamic contrast-enhanced magnetic resonance imaging features were extracted, including size, shape and texture. Using recursive feature elimination and gradient boosting machine, a radiomics model was established to predict HLA-DQA1 expression. Receiver operating characteristic (ROC) curves, precision-recall curves, calibration curves, and decision curves were used for model evaluation.

**Results:**

The HHD group had better survival outcomes. The differentially expressed genes in the HHD group were significantly enriched in oxidative phosphorylation (OXPHOS) and estrogen response early and late signalling pathways. The radiomic score (RS) output from the model was associated with HLA-DQA1 expression. The area under the ROC curves (95% CI), accuracy, sensitivity, specificity, positive predictive value, and negative predictive value of the radiomic model were 0.866 (0.775–0.956), 0.825, 0.939, 0.7, 0.775, and 0.913 in the training set and 0.780 (0.629–0.931), 0.659, 0.81, 0.5, 0.63, and 0.714 in the validation set, respectively, showing a good prediction effect.

**Conclusions:**

High HLA-DQA1 expression is associated with a better prognosis in breast cancer. Quantitative radiomics as a noninvasive imaging biomarker has potential value for predicting HLA-DQA1 expression.

## Key points

1. High HLA-DQA1 expression is associated with a better prognosis in breast cancer.
2. The differentially expressed genes in the HHD group were significantly enriched in OXPHOS and estrogen response early and late signalling pathways.
3. The expression of HLA-DQA1 in breast cancer could be predicted with the radiomics model.

## Introduction

Breast cancer is the most common malignant tumour and the second most common cause of cancer-related death in women worldwide [1]. The Cancer Genome Atlas Network revealed large differences between different breast cancer subtypes [2]. The polymorphism of human leukocyte antigen (HLA) is associated with the risk for and progression of various autoimmune and malignant diseases [3]. HLA-DQA1 belongs to the alpha chain of human major histocompatibility complex class II (MHC-II) and plays a decisive role in the pathogenesis of breast cancer [4]. In addition, the HLA-DQA1 gene predicts hepatotoxicity risk in breast cancer treated with epidermal growth factor receptor (EGFR) inhibitors [5].

Dynamic contrast-enhanced magnetic resonance imaging (DCE-MRI) is the most accurate method for diagnosing and evaluating breast cancer, with modest sensitivity and specificity (approximately 70%) [6]. Radiomics based on DEC-MRI is currently a focus of cancer research and has the advantages of being noninvasive, rapid, affordable and repeatable. It can reflect the underlying molecular and genotypic basis of the tissue, providing unprecedented insights and facilitating a deeper understanding of breast cancer development and progression [7–11]. However, the ability of radiomics to assess the expression of HLA-DQA1 in breast cancer is unclear.

In the present study, the relationship between HLA-DQA1 expression and breast cancer prognosis was explored, and then the potential molecular mechanisms of different HLA-DQA1 expression groups were analysed. Finally, a radiomics model that can predict HLA-DQA1 expression was established as a new practical imaging biomarker for breast cancer prognosis.

## Methods and materials

### Dataset acquisition

The data analysed in this retrospective study were obtained from The Cancer Genome Atlas (TCGA, https://portal.gdc.cancer.gov) and The Cancer Imaging Archive (TCIA, http://www.cancerimagingarchive.net/) databases. Ethical approval was granted by the institutional review board of the TCIA host institution.

Dataset A: Genetic data were obtained from the TCGA-BRCA cohort, comprising 1097 patients. After excluding 51 patients with a survival time of less than 30 days or with missing survival data, 51 patients with incomplete clinical data, 12 male patients, and 21 patients with primary tumours in other sites, 962 patients were included in the study. According to the expression of HLA-DQA1, the patients were divided into a high HLA-DQA1 expression group (HHD group) (n = 492) and a low HLA-DQA1 expression group (LHD group) (n = 470) by the cut-off value 4.105 of the median expression level, and statistical description was performed.

Dataset B: Imaging data were obtained from the TCIA-BRCA cohort, comprising 137 patients. After excluding 29 patients with poor image quality or postoperation and 4 without gene expression data available, 104 patients were included in the imaging genomics analysis. The dataset was randomly divided into a training set (n = 63) and a validation set (n = 41) at a ratio of 6:4, and statistical description was performed.

### Bioinformatics analysis

#### Data processing

RNAseq data in the format of transcripts per million reads (TPM) for both TCGA and GTEx were downloaded from UCSC Xena (https://xenabrowser.net/datapages/), and then all 179 normal tissue data from GTEx were extracted. The RNAseq data were processed uniformly by the Toil program and then analysed after log2 transformation [12].

#### Differential expression analysis

Breast cancer tissue data from TCGA-BRCA and normal tissue data from GTEx were extracted and log-transformed. The differences in HLA-DQA1 expression among the HHD, LHD, and normal groups were compared.

#### Functional enrichment analysis

Gene set enrichment analyses (GSEA) for the KEGG (c2.cp.kegg.v7.5.1.symbols.gmt) and Hallmark (h.all.v7.5.1.symbols.gmt) gene sets were performed on each sample using the "clusterProfiler" package in R. Results with P values less than 0.05 and false discovery rate (FDR) values less than 0.25 were considered significant.

#### Survival analysis

The “cmprsk”, “survival” and “forestplot” packages of R were applied. Kaplan‒Meier survival curves and Cox proportional hazards regression models were used to calculate the overall survival (OS) time. The log-rank test was used to test the significance of survival between groups. Univariate and multivariate Cox proportional hazards regression models were conducted to evaluate the effect of HLA-DQA1 on survival outcomes. The effect of HLA-DQA1 in covariate subgroups was explored using stratified analyses.

### Radiomic analysis

#### Imaging data

DCE-MRI images from dataset B were obtained using T1-weighted spoiled gradient echo sequences and gadolinium contrast medium, with sagittal or axial views. The average resolution was 0.7 mm (range 0.5–0.8). The slice thickness of the MRI sequence was approximately 2 mm, and the image size was 512 × 512 pixels or 256 × 256 pixels. In order to reduce the effect due to the variability of different types of image, spatial resampling and image intensity normalization were applied.

#### Imaging segmentation and image feature extraction

Lesion segmentation was performed in 3D-Slicer (v4.10.2; https://www.slicer.org/). Volumes of interest (VOIs) were manually delineated layer by layer by a double-blind radiologist (with 10 years of experience in radiology) independently. Another double-blind radiologist (with 5 years of experience in radiology) randomly selected 30 cases for secondary delineation. VOI delineation followed these rules: (1) Selected the phase of MRI image with the most obvious lesion enhancement (the signal ratio of lesion to background) to depict the tumour area. (2) Considered all images comprehensively when delineating lesions. (3) Contrasted bilaterally to identify the mass, structural and signal changes, abnormal enhancement. (4) Determined the location and boundary of the lesions by adjusting the appropriate window width.

Image feature extraction was performed in PyRadiomics. A total of 107 radiomics features were extracted, including tumour size (such as volume, surface area, maximum three-dimensional diameter and long axis length), morphology (such as elongation, flatness and sphericity) and texture (such as energy, entropy, kurtosis, skewness, grey level size zone matrix (GLSZM), grey level dependence matrix (GLDM), grey level cooccurrence matrix (GLCM) and grey level run length matrix (GLRLM).

Imaging features were normalized separately for each scanner type and protocol to minimize the impact of differences between different scanners and scanning protocols (z score normalization, mean = 0 and standard deviation = 1). The consistency between the two radiologists was compared, and the features with intraclass correlation coefficients (ICCs) greater than or equal to 0.75 were applied in the next step of the study.

#### Feature selection and radiomics model establishment

The “caret”, “pROC”, “measures”, “rms”, “rmda”, “ggpubr” and “Resource Selection” R packages were applied. Features were screened by recursive feature elimination (RFE), and the radiomic model for HLA-DQA1 prediction was established by a gradient boosting machine (GBM) algorithm. The algorithm's task was to find the best performing feature set by maximizing the model's accuracy on the training set.

#### Radiomics model evaluation

The model's effectiveness was evaluated in the training and validation sets. Receiver operating characteristic (ROC) curves and precision-recall (PR) curves were used for model evaluation. The evaluation indexes included accuracy (ACC), specificity (SPE), sensitivity (SEN), positive predictive value (PPV) and negative predictive value (NPV). The calibration of the model was evaluated by drawing a calibration curve. The Hosmer‒Lemeshow test and BrierScore were used to quantify the comprehensive performance of the model. The decision curve (DCA) was drawn to demonstrate the clinical benefit of the model. The radiomic score (RS) for the prediction of HLA-DQA1 expression was obtained from the radiomics model and compared between groups.

### Statistical analysis

R software (v4.1.0, https://cran.r-project.org/) was used for the statistical analyses. Imaging features were extracted using PyRadiomics (python, v3.7.6, https://www.python.org/downloads/). The above software is open source. The Shapiro‒Wilk test and one-way ANOVA were used for the normal distribution of continuous variable data. Variables conforming to a normal distribution and homogeneity of variance were tested by Student's t test; otherwise, the Mann‒Whitney U test was used. The χ^2^ test or Fisher's exact test was used for categorical variables. The Kruskal‒Wallis or Wilcoxon test was used for measurement or ordinal data. Spearman's test was used to assess the correlation between variables. The criterion for a statistically significant difference was P < 0.05.

## Result

### Bioinformatics analysis

The clinical characteristics of the 962 patients in Dataset A are shown in Table 1. There were significant statistically differences in HER2 status, ER status, chemotherapy, histological type and overall survival between groups.

**Table 1:** Clinical characteristics of 962 breast cancer patients in the TCGA-BRCA cohort

Variables	Total (n = 962)	LHD-group (n = 470)	HHD-group (n = 492)	*p*
HLA-DQA1 expression, (${\overline{\text{X}}} \pm {\text{SD}}$)*				< 0.001
	4.147 ± 1.220	3.146 ± 0.744	5.103 ± 0.713	
Age, n (%)				0.091
~ 59	521 (54)	241 (51)	280 (57)	
60~	441 (46)	229 (49)	212 (43)	
PR_status, n (%)				0.189
Negative	307 (32)	140 (30)	167 (34)	
Positive	655 (68)	330 (70)	325 (66)	
HER2_status, n (%)				0.033
Negative	512 (53)	233 (50)	279 (57)	
Equivocal/Indeterminate	304 (32)	167 (36)	137 (28)	
Positive	146 (15)	70 (15)	76 (15)	
ER_status, n (%)				0.001
Negative	216 (22)	84 (18)	132 (27)	
Positive	746 (78)	386 (82)	360 (73)	
Radiotherapy, n (%)				0.319
No	456 (47)	231 (49)	225 (46)	
Yes	506 (53)	239 (51)	267 (54)	
Chemotherapy, n (%)				0.006
No	426 (44)	230 (49)	196 (40)	
Yes	536 (56)	240 (51)	296 (60)	
T_stage, n (%)				0.124
T1	258 (27)	126 (27)	132 (27)	
T2	547 (57)	256 (54)	291 (59)	
T3/T4	157 (16)	88 (19)	69 (14)	
N_stage, n (%)				0.785
N0	447 (46)	221 (47)	226 (46)	
N1/N2/N3/NX	515 (54)	249 (53)	266 (54)	
M_stage, n (%)				0.482
M0	797 (83)	394 (84)	403 (82)	
M1/MX	165 (17)	76 (16)	89 (18)	
Histological_type, n (%)				0.006
ILC	187 (19)	77 (16)	110 (22)	
IDC	682 (71)	336 (71)	346 (70)	
Other	93 (10)	57 (12)	36 (7)	
OS, n (%)				0.004
Alive	829 (86)	389 (83)	440 (89)	
Dead	133 (14)	81 (17)	52 (11)	
OS.time, Median(Q1, Q3)				
	29.4 (16.3, 59.4)	29.4 (15.9, 55.8)	29.7 (16.6, 64.0)	0.404

*HHD group* high HLA-DQA1 expression group, *LHD group* low HLA-DQA1 expression group, *PR* progesterone receptor, *ER* estrogen receptor, *HER2* human epidermal growth factor receptor 2, *ILC* infiltrating lobular carcinoma, *IDC* infiltrating ductal carcinoma, *OS* overall survival

**Fig. 1:**
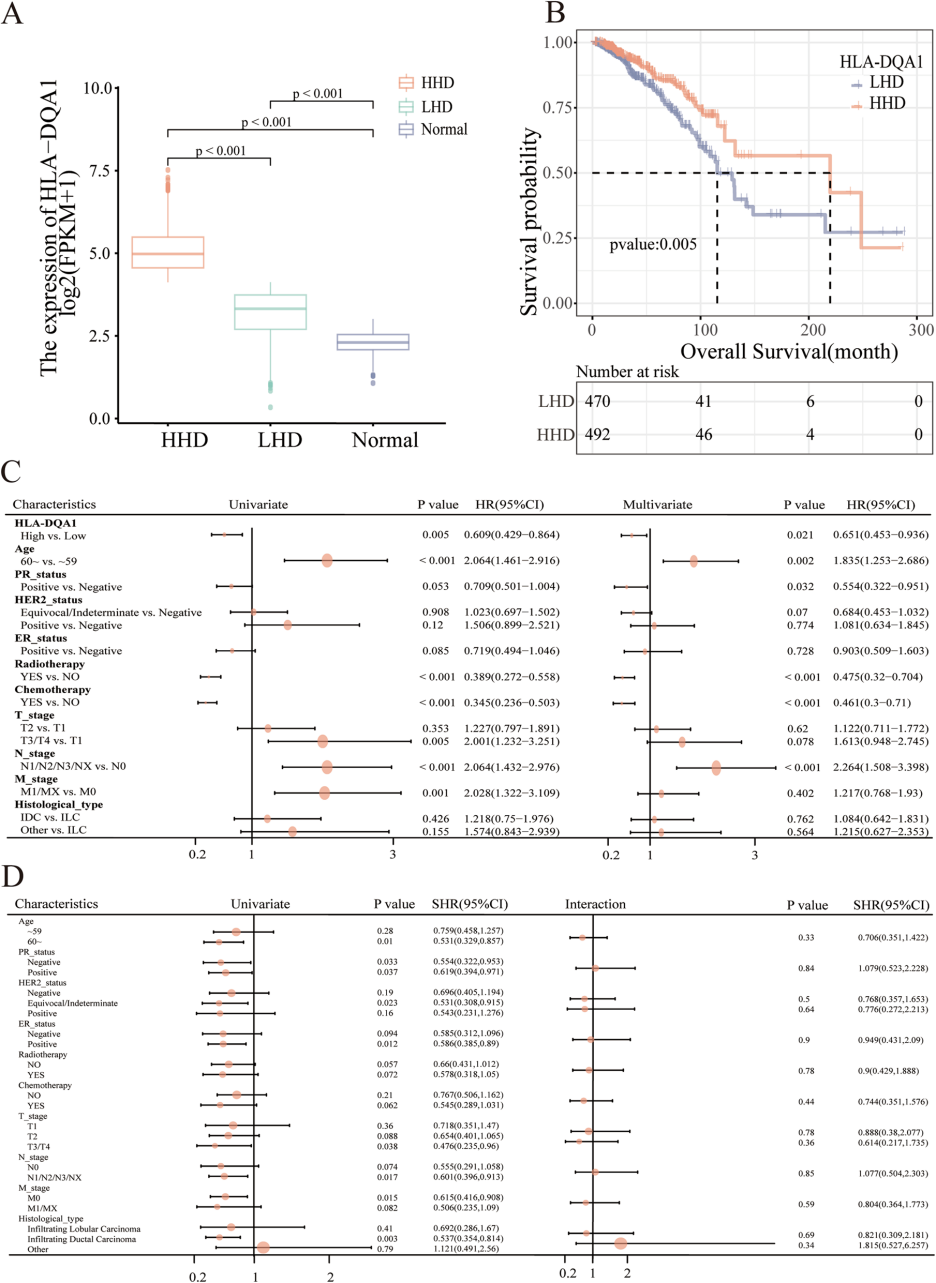
Bioinformatics analysis results. **A** Comparison of HLA expression among the HHD, LHD and normal groups. **B** Kaplan‒Meier curves for patients in the HHD and LHD groups. **C** Univariate and multivariate Cox analyses of clinicopathological factors and key genes. **D** Subgroup analyses showed no interaction between the main variable HLA-DQA1 (high vs. low) and each covariate

The expression of HLA-DQA1 in the HHD group and LHD group was higher than that in normal tissue, and the differences were statistically significant (Fig. 1A).

In the survival analysis, the HHD group had better survival outcomes than the LHD group (Fig. 1B). The median survival time of the LHD group was 115.4 months, and that of the HHD group was 217.77 months. Higher HLA-DQA1 expression was associated with longer OS durations (P = 0.005).

In the univariate Cox proportional hazards regression analysis, HLA-DQA1 (HR (95% CI) = 0.609 (0.429–0.864), P = 0.005), radiotherapy (HR (95% CI) = 0.389 (0.272–0.558), P < 0.001) and chemotherapy (HR (95% CI) = 0.345 (0.236–0.503), P < 0.001) were protective factors for OS outcomes. After multivariate adjustment, HLA-DQA1 (HR (95% CI) = 0.651 (0.453–0.936), P = 0.021), PR (HR (95% CI) = 0.554 (0.322–0.951), P = 0.032), radiotherapy (HR (95% CI) = 0.475 (0.32–0.704), P < 0.001), and chemotherapy (HR (95% CI) = 0.461 (0.3–0.71), P < 0.001) were statistically significant protective factors for OS outcomes, while age and N stage were risk factors. Details are shown in Fig. 1C.

In the subgroup analysis (Fig. 1D), although high HLA-DQA1 expression was a protective factor in subgroups of age > 60 years, all PR status, ER positivity, T stage 3/4, N stage 1–3, M stage 0, and IDC (P < 0.05), the P values for interaction tests between subgroups of each covariate were all greater than 0.05. Thus, there was no significant interaction between high HLA-DQA1 expression and each covariate; in other words, the effect of high HLA-DQA1 expression on OS outcomes was similar across subgroups of each covariate.

**Fig. 2:**
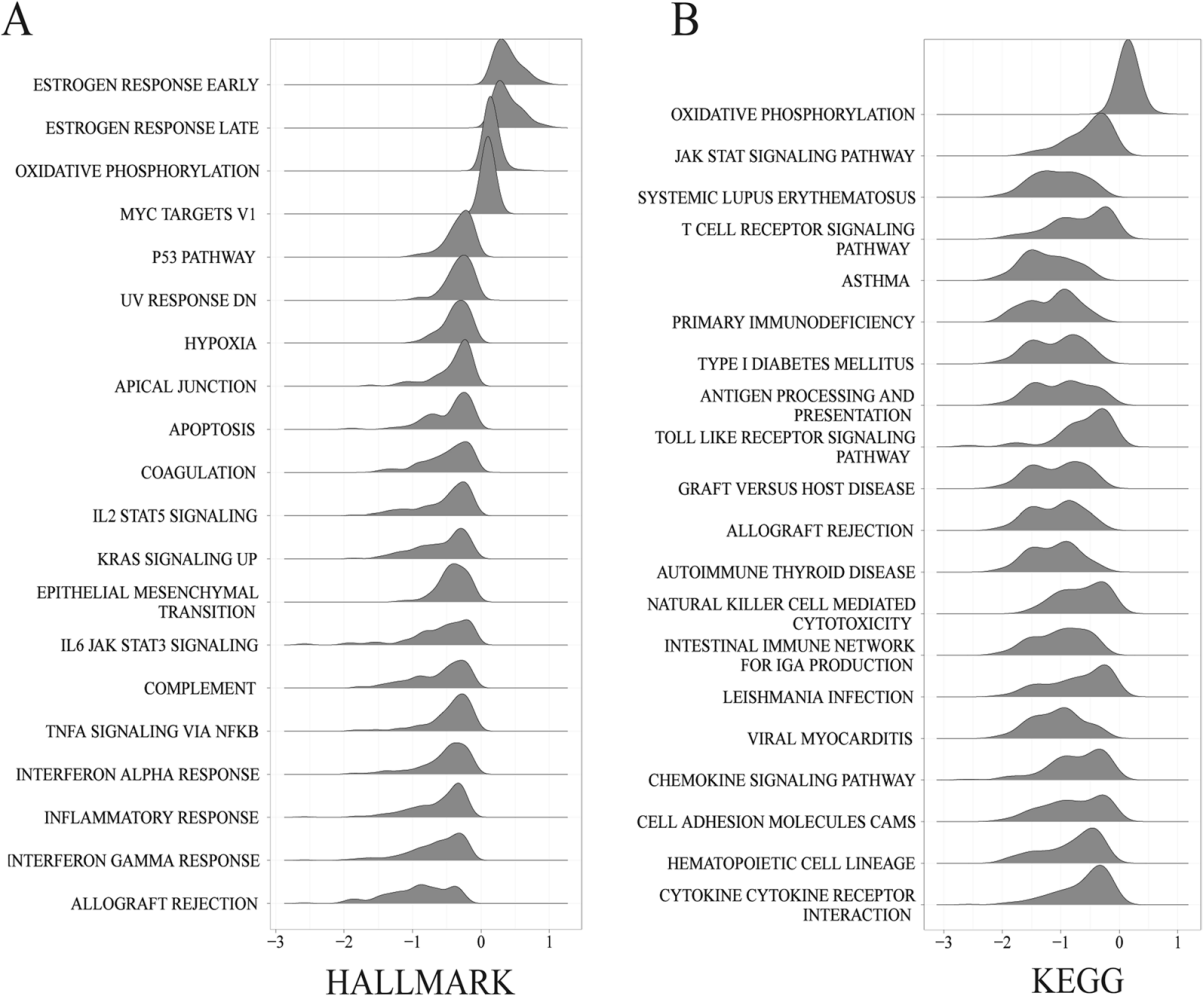
Visualization results of the top 20 pathways for the Hallmark and KEGG gene sets by gene set enrichment analysis

The KEGG gene set enrichment analysis (Fig. 2) showed that the differentially expressed genes in the HHD group were significantly enriched in the oxidative phosphorylation (OXPHOS) signalling pathway. Hallmark gene set enrichment analysis showed that the differentially expressed genes in the HHD group were significantly enriched in the estrogen response early and late signalling pathways.

### Radiomic analysis

The clinical characteristics of the 104 patients in Dataset B are shown in Table 2. There was no significant statistical difference in clinical characteristics between the training set and the validation set, indicating that the baseline of patients was similar and the division did not cause significant bias.

**Table 2:** Clinical characteristics of 104 breast cancer patients in the TCIA-BRCA cohort

Variables	Total (n = 104)	Training (n = 63)	Validation (n = 41)	p
HLA-DQA1, n (%)				1
Low	50 (48)	30 (48)	20 (49)	
High	54 (52)	33 (52)	21 (51)	
Age, n (%)				0.251
~ 59	69 (66)	45 (71)	24 (59)	
60~	35 (34)	18 (29)	17 (41)	
PR_status, n (%)				1
Negative	25 (24)	15 (24)	10 (24)	
Positive	79 (76)	48 (76)	31 (76)	
HER2_status, n (%)				0.479
Equivocal/Indeterminate	31 (30)	19 (30)	12 (29)	
Negative	58 (56)	37 (59)	21 (51)	
Positive	15 (14)	7 (11)	8 (20)	
ER_status, n (%)				0.232
Negative	17 (16)	13 (21)	4 (10)	
Positive	87 (84)	50 (79)	37 (90)	
Radiotherapy, n (%)				0.15
No	31 (30)	15 (24)	16 (39)	
Yes	73 (70)	48 (76)	25 (61)	
Chemotherapy, n (%)				0.6
No	27 (26)	18 (29)	9 (22)	
Yes	77 (74)	45 (71)	32 (78)	
T_stage, n (%)				0.553
T1	40 (38)	22 (35)	18 (44)	
T2	59 (57)	37 (59)	22 (54)	
T3/T4	5 (5)	4 (6)	1 (2)	
N_stage, n (%)				0.519
N0	53 (51)	30 (48)	23 (56)	
N1/N2/N3/NX	51 (49)	33 (52)	18 (44)	
M_stage, n (%)				1
M0	96 (92)	58 (92)	38 (93)	
M1/MX	8 (8)	5 (8)	3 (7)	
Histological_type, n (%)				0.24
IDC	89 (86)	51 (81)	38 (93)	
ILC	11 (11)	9 (14)	2 (5)	
Other	4 (4)	3 (5)	1 (2)	
OS, n (%)				1
Alive	103 (99)	62 (98)	41 (100)	
Dead	1 (1)	1 (2)	0 (0)	
OS.time, Median (Q1,Q3)				0.727
	40.53 (24.74, 64.03)	40.1 (25.15, 64.23)	40.97 (24.27, 60)	

*PR* progesterone receptor, *ER* estrogen receptor, *HER2* human epidermal growth factor receptor 2, *ILC* infiltrating lobular carcinoma, *IDC* infiltrating ductal carcinoma, *OS* overall survival

**Fig. 3:**
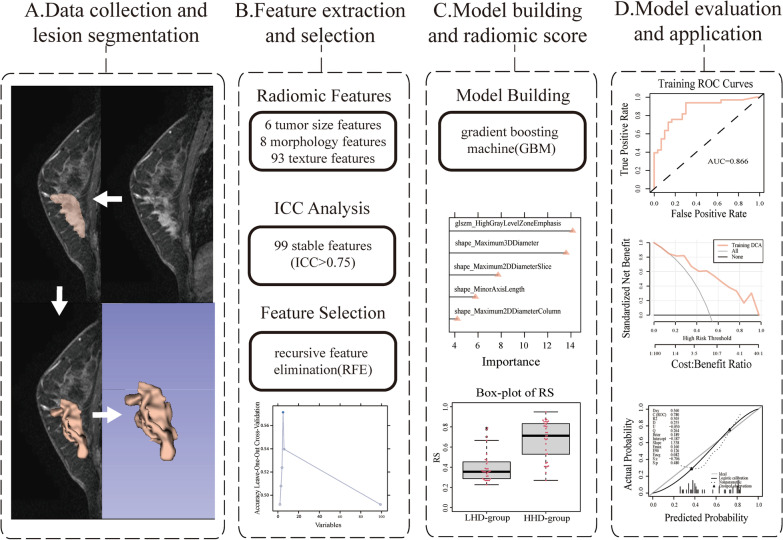
Graphical flowchart of the radiomics analysis. **A** Imaging data collection and lesion segmentation. **B** Feature extraction using PyRadiomics and feature selection using recursive feature elimination (RFE). **C** Modelling by the gradient boosting machine (GBM) algorithm and outputting the radiomics score (RS). **D** Model evaluation and application using ROC curves, the decision curve and the calibration curve

**Fig. 4:**
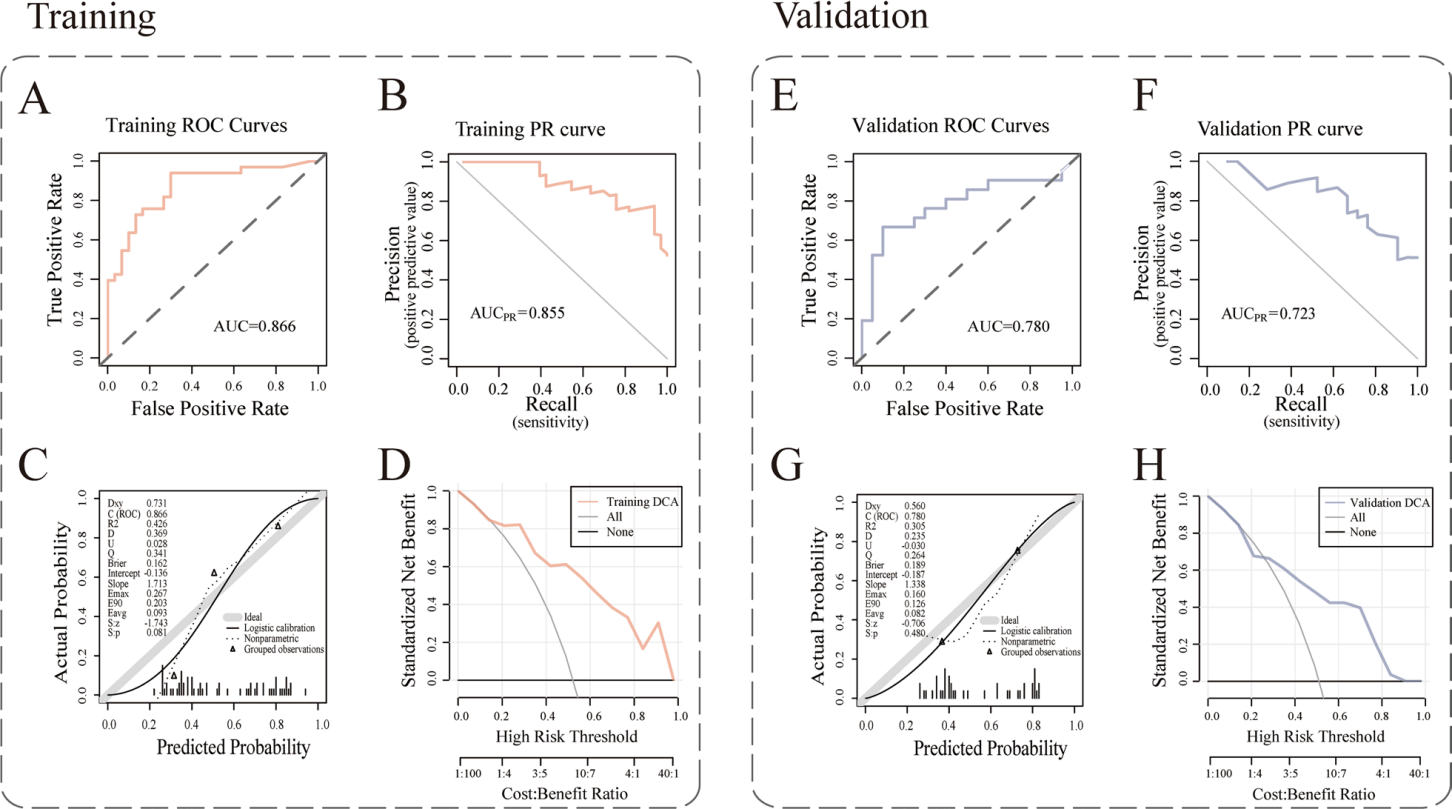
Results of the evaluation of the RFE-GBM radiomics model. The AUC of ROC curves and the AUC of precision-recall curve (AUC_PR_) of the model were 0.866 and 0.855, respectively, in the training set (**A** and **B**) and 0.780 and 0.723, respectively, in the validation set (**E** and **F**). The calibration curve and Hosmer‒Lemeshow test (**C** and **G**) showed that the prediction probability of high HLA-DQA1 expression was consistent with the true value, and P > 0.05 indicated good consistency. The DCA curve (**D** and **H**) showed that the model had clinical practicability within a certain range

The process of radiomics analysis was depicted in Fig. 3. The median ICC value of the radiomics features extracted by the two radiologists was 0.914, and there were 99 radiomics features with an ICC value greater than or equal to 0.75. After screening these features by RFE, five features were used to establish the RFE-GBM radiomic model, namely, original_shape_Maximum2DdiameterSlice, original_glszm_HighGrayLevelZoneEmphasis, original_shape_Maximum3DDiameter, original_shape_MinorAxisLength, and original_shape_Maximum2DDiameterColumn. As shown in Fig. 4, the AUC of ROC curves (95% CI), accuracy, sensitivity, specificity, positive predictive value, negative predictive value and BrierScore of the model were 0.866 (0.775–0.956), 0.825, 0.939, 0.7, 0.775, 0.913 and 0.162 in the training set and 0.780 (0.629–0.931), 0.659, 0.81, 0.5, 0.63, 0.714 and 0.189 in the validation set, respectively, showing a good prediction effect. The RS value of HLA-DQA1 expression predicted by the model was significantly different between groups (P < 0.05). Higher HLA-DQA1 expression was associated with a higher RS value.

## Discussion

HLA, a subtype of MHC class II molecule, is the most diverse molecular structural region expressed in humans. Low HLA-DQA1 expression was associated with poor prognosis in hepatocellular carcinoma, lung cancer, and soft tissue sarcoma patients as its reduction indicated the presence of an immunosuppressive microenvironment and invasive disease [13–15]. The results of the present study suggested that low HLA-DQA1 expression was associated with poor prognosis in breast cancer patients. As shown at baseline, there were more HER2-positive and ER-negative patients, and more patients receiving chemotherapy in the HHD group than in the LHD group. Although more aggressive, HER2-positive breast cancers respond better to chemotherapy, especially when combined with trastuzumab and pertuzumab [16, 17], which may be the reason for the better prognosis in the HHD group. In the multifactor analysis of the present study, this opinion was supported by the protective effect of chemotherapy and the absence of HER2 as a risk factor.

The differences between the HHD and LHD groups indicated that ER and HER2 receptors were epigenetically related to the expression of HLA-DQA1. Generally speaking, HER2 is thought to be mainly involved in the Ras/RAF/MEK/ERK pathway for cell proliferation and the PI3K/Akt/mTOR pathway for cell survival [18–20]. In the present study, the differentially expressed genes with high HLA-DQA1 expression were enriched in the OXPHOS, estrogen response early and estrogen response late signalling pathways. The enrichment of the estrogen response early and late signalling pathway may be caused by feedback regulation of ER-negative. Peroxisome proliferator-activated receptor gamma co-activator 1 alpha (PGC1α) promotes metastasis by mediating mitochondrial biogenesis and OXPHOS in cancer cells. Estrogen-related receptor α is a cofactor of PGC1α and an essential factor for nuclear mitochondrial gene transcription and mitochondrial biogenesis [21]. These results suggested that HLA-DQA1 might inhibit the growth of breast cancer by regulating mitochondrial metabolism through the PGC1α pathway. Confirmation of this relationship requires further experiments.

However, high HLA-DQA1 expression is not always a protective factor in cancer. Shen et al. [22] found that the expression of HLA-DQA1 was upregulated in esophageal squamous cell carcinoma and associated with poor prognosis and shorter survival times. The higher the expression level of HLA-DQA1 is, the larger the tumour, and the higher the probability of familial disease will be. There are several possible causes: tumours are not sensitive to targeted therapy, tumour proliferation is not regulated by mitochondrial metabolism, or high HLA expression is associated with other potential pathways. Therefore, in further studies, subgroup analysis of breast cancers that do not respond well to chemotherapy may yield new findings.

In a study of 47 triple-negative breast cancer (TNBC) patients by RNA sequencing, HLA-DQA1 was associated with improved progression-free survival [23]. By means of proteomics, Asleh K et al. [24] showed that high expression of HLA-DQA1 as a single tumour biomarker showed significantly better recurrence-free survival rates. The results of survival analysis of different HLA-DQA1 expression levels in the present study were consistent with the previous studies. Unlike previous studies, this study was not limited to single molecular typing of breast cancer and used a different approach radiomics to predict HLA-DQA1 expression. Anchoring radiomics and HLA-DQA1 expression simplifies the HLA-DQA1 assessment procedure and reduces costs, enabling personalized, precise medicine.

The central assumption of radiomics is that medical images contain information that reflects pathophysiology that can be revealed by quantitative image analysis. Tumour heterogeneity has been suggested to be related to Ras signalling through the analysis of tumour subregions and can be detected by radiomics [25]. Our results also demonstrated that radiomics could accurately and noninvasively estimate tumour HLA-DQA1 expression with AUCs of 0.866 and 0.780 in the training and validation groups, respectively. Radiomics provides substantial data related to microstructure heterogeneity, tumour microenvironment, and epigenetics for mining.

Zhu et al. [26] found that the quantitative MRI features of tumours (such as tumour size, shape, resection margin, and haemodynamics) correlated with their corresponding molecular spectra (such as DNA mutations, miRNA expression, protein expression, pathway gene expression, and copy-number variations). In the present study, the morphological features and GLCM features had the best performance in revealing the biological characteristics of tumours. The morphological features of tumours were related to invasiveness, and GLCM features were related to heterogeneity. Moon et al. [27] found that DCE-MRI radiomics features based on wavelet transform GLCM had a better ability to identify TP53 and PIK3CA mutations than morphological features in breast cancer. The more malignant the tumour is, the larger the volume, the more common the marginal infiltration and high heterogeneity will be. In the present study, the selected features contained more morphological features and had better performance than GLCM features.

Although we used public databases and open-source software to make our results generalizable, several limitations remain. First, this study was retrospective, and genetic data were not routinely available for most breast cancer patients; thus, the sample size was small and might not be sufficiently representative. Genetic testing is expensive and complex, limiting large-scale imaging genomics studies. Second, the radiomic features used in our study might not be comprehensive; given the sample size of the study cohort, we used only the conventional feature sets from open-source software. It is necessary to enrich the feature types in further study. Finally, even with normalization, different scanners, scanning schemes, and manual segmentation still affected the extraction of radiomics features. A prospective cohort study is required to be conducted.

## Conclusion

In conclusion, the present study indicated that high HLA-DQA1 expression is associated with a better prognosis in breast cancer patients and the differentially expressed genes are enriched in the OXPHOS, estrogen response early and estrogen response late signalling pathways. Although the above results still need to be validated in prospective cohort studies, quantitative radiomics has potential value as a noninvasive imaging biomarker for predicting HLA-DQA1 expression.

## Data Availability

The datasets generated and/or analysed during the current study are available in The Cancer Genome Atlas (TCGA, https://portal.gdc.cancer.gov/) and The Cancer Imaging Archive (TCIA, http://www.cancerimagingarchive.net/) databases.

## Abbreviations

DCE-MRI: Dynamic contrast-enhanced magnetic resonance imaging
HLA: Human leukocyte antigen
MHC-II: Major histocompatibility complex class II
EGFR: Epidermal growth factor receptor
TCGA: The Cancer Genome Atlas
TCIA: The Cancer Imaging Archive
TPM: Transcripts Per Millionreads
GSEA: Gene set enrichment analysis
FDR: False discovery rate
OS: Overall survival
VOIs: Volumes of interest
GLSZM: Grey level size zone matrix
GLDM: Gray level dependence matrix
GLCM: Gray level cooccurrence matrix
GLRLM: Gray level run length matrix
ICCs: Intraclass correlation coefficient
RFE: Recursive feature elimination
GBM: Gradient boosting machine
ACC: Accuracy
SPE: Specificity
SEN: Sensitivity
PPV: Positive predictive value
NPV: Negative predictive value
DCA: Decision curve
AUC: Area under curve
RS: Radiomic score
PR: Progesterone receptor
ER: Estrogen receptor
HER2: Human epidermal growth factor receptor 2
IDC: Infiltrating ductal carcinoma
Ras/RAF/MEK/ERK: Ras/Raf kinases/Mitogen-activated protein kinase kinase/ Extracellular signal regulated kinases
PI3K/Akt/mTOR: Phosphoinositide 3-Kinase/Akt/Mammalian target of Rapamycin
OXPHOS: Oxidative phosphorylation
PGC1α: Peroxisome proliferator-activated receptor gamma co-activator 1 alpha
TNBC: Triple-negative breast cancer
